# An immuno-assay to quantify influenza virus hemagglutinin with correctly folded stalk domains in vaccine preparations

**DOI:** 10.1371/journal.pone.0194830

**Published:** 2018-04-04

**Authors:** Madhusudan Rajendran, Weina Sun, Phillip Comella, Raffael Nachbagauer, Teddy John Wohlbold, Fatima Amanat, Ericka Kirkpatrick, Peter Palese, Florian Krammer

**Affiliations:** 1 Department of Microbiology, Icahn School of Medicine at Mount Sinai, New York, NY, United States of America; 2 Graduate School of Biomedical Sciences, Icahn School of Medicine at Mount Sinai, New York, NY, United States of America; 3 Department of Medicine, Icahn School of Medicine at Mount Sinai, New York, NY, United States of America; Deutsches Primatenzentrum GmbH - Leibniz-Institut fur Primatenforschung, GERMANY

## Abstract

The standard method to quantify the hemagglutinin content of influenza virus vaccines is the single radial immunodiffusion assay. This assay primarily relies on polyclonal antibodies against the head domain of the influenza virus hemagglutinin, which is the main target antigen of influenza virus vaccines.

Novel influenza virus vaccine candidates that redirect the immune response towards the evolutionary more conserved hemagglutinin stalk, including chimeric hemagglutinin and headless hemagglutinin constructs, are highly dependent on the structural integrity of the protein to present conformational epitopes for neutralizing antibodies. In this study, we describe a novel enzyme-linked immunosorbent assay that allows quantifying the amount of hemagglutinin with correctly folded stalk domains and which could be further developed into a potency assay for stalk-based influenza virus vaccines.

## Introduction

The traditional method to measure the potency of influenza virus vaccines is the single radial immunodiffusion (SRID) assay [1, 2]. This assay has been accepted by the United States Food and Drug Administration (FDA) since 1978 for the measurement of the hemagglutinin (HA) content of influenza vaccines based on antibodies to the HA globular head domain [3]. Antibodies against the globular head domain are generally hemagglutination inhibition (HI) active, and the HI titers are an established correlate of protection [4]. Furthermore, HA amounts quantified via SRID assay have been linked to *in vivo* potency as measured by increases of HI titers post vaccination [5–7]. Recently, influenza virus vaccine candidates that are based on inducing antibodies against the conserved stalk domain of the HA have been developed [8, 9]. Neutralizing antibodies against the stalk domain of the HA are rare but can be elicited using specific vaccination regimens, e.g. using chimeric HAs (cHA) or headless HA constructs [8, 10, 11]. Importantly, the majority of neutralizing anti-stalk antibodies bind to conformational epitopes that can be damaged or completely destroyed by physical or chemical stress including freeze-thawing, high temperatures or low pH [12–16]. The development of stalk-based vaccines therefore requires an assay that measures the content of correctly folded HA in a vaccine preparation and which can ultimately be linked to *in vivo* potency. Here, we report a capture enzyme-linked immunosorbent assay (ELISA) that can be used to detect and quantitatively measure HA with conformationally intact stalk epitopes.

## Materials and methods

### Virus rescue and generation of virus preparations

Viruses expressing different chimeric HAs (cHA, see Table 1) were rescued through reverse genetics by the use of an eight-plasmid system [17]. Briefly, the cHA and neuraminidase (NA) rescue plasmids were generated by using In-Fusion cloning (Clontech). The packaging signals for the HA and NA genomic segments were derived from the respective A/Puerto Rico/8/34 (PR8) virus genomic segments. The viruses used in this study expressed the NA from A/California/04/2009 (Cal09) and the six internal segments (PB2, PB1, PA, NP, M and NS) were derived from PR8 virus. Details about the cHA expressing viruses are listed in Table 1. All segments were cloned into a pDZ rescue vector that expresses a negative-sense genomic transcript (vRNA) driven by a Pol-I promoter and a positive sense transcript of the viral gene driven by a Pol-II promotor (mRNA). To generate virus, 293T cells were transfected with 1μg of plasmids for each one of the eight viral segments using TransIT-LT1 (Mirus). After 48h, cells and supernatants were collected and injected into 8-day old embryonated chicken eggs that were incubated at 37˚C [17, 18]. Forty-eight hours after injection, the eggs were cooled down to 4°C for 4–12 hours, harvested and clarified by low speed centrifugation (1500rpm, 10min). Viral rescue cultures were initially screened by performing hemagglutination assays. Positive virus cultures were plaque purified and expanded in embryonated chicken eggs. Virus titers were determined by plaque assay on Madin Darby canine kidney (MDCK) cells in the presence of tosyl phenylalanyl chloromethyl ketone (TPCK)-treated trypsin. The following wild type isolates/sequences were used in the study: PR8 (H1N1), Cal09 (pandemic H1N1, 6:2 re-assortant with PR8 backbone), A/Dominican Republic/7293/13 (pandemic H1N1, DR13), A/Netherlands/602/09 (pandemic H1N1, NL09), A/Hong Kong/2014 (H3N2, HK14), A/Perth/16/2009 (H3N2, Perth09), A/Victoria/2011 (H3N2, Vic11), A/duck/Czech/1956 (H4N6, dCZ56), A/Vietnam/1203/04 (H5N1, VN04), A/mallard/Sweden/24/02 (H8N4, mSW02), A/shoveler/Netherlands/18/99 (H11N9, sNL99) and A/mallard/Interior Alaska/7MP0167/07 (H12N5, mIA07). Chimeric HA expressing viruses are described below, viruses used for the longitudinal stability study are listed in Table 1.

**Table 1:** Viruses tested for stability during storage at 4°C and 27°C.

Virus	HA and NA	Comments
**cH5/1_Cal09_N1**	H5 head domain from VN04, H1 stalk domain and NA from Cal09	wild type stalk (E374)
**cH5/1_Cal09ss_N1**	H5 head domain from VN04, H1 stalk domain and NA from Cal09	E374K mutation
**cH5/1_DR13_N1**	H5 head domain from VN04, H1 stalk domain from DR13 and NA from Cal09	wild type stalk (K374)
**cH8/1_Cal09_N1**	H8 head domain from mSW02, H1 stalk domain and NA from Cal09	wild type stalk (E374)
**cH8/1_Cal09ss_N1**	H8 head domain from mSW02, H1 stalk domain and NA from Cal09	E374K mutation
**cH8/1_DR13_N1**	H8 head domain from mSW02, H1 stalk domain from DR13 and NA from Cal09	wild type stalk (K374)
**cH11/1_Cal09_N1**	H11 head domain from sNL99, H1 stalk domain and NA from Cal09	wild type stalk (E374)
**cH11/1_Cal09ss_N1**	H11 head domain from sNL99, H1 stalk domain and NA from Cal09	E374K mutation
**cH11/1_DR13_N1**	H11 head domain from sNL99, H1 stalk domain from DR13 and NA from Cal09	wild type stalk (K374)
**cH12/1_Cal09_N1**	H12 head domain from mIA07, H1 stalk domain and NA from Cal09	wild type stalk (E374)
**cH12/1_Cal09ss_N1**	H12 head domain from mIA07, H1 stalk domain and NA from Cal09	E374K mutation
**cH12/1_DR13_N1**	H12 head domain from mIA07, H1 stalk domain from DR13 and NA from Cal09	wild type stalk (K374)
**Cal09**	wild type HA and NA from Cal09	wild type stalk (E374)
**DR13**	wild type HA and NA from DR13	wild type stalk (K374)
**cH4/3N2**	H4 head from dCZ56, H3 stalk from Perth09, NA from Vic11	group 2 HA

Purified virus preparations were used in capture ELISA. In order to purify the viruses, eggs were injected with 500 plaque forming units (PFU) of virus. After an incubation period of forty-eight hours at 37˚C, eggs were cooled down to 4°C for 4–12 hours, allantoic fluid was harvested and cleared by low speed centrifugation at 3,000 relative centrifugal force (rcf). The pooled allantoic fluid was ultracentrifuged at 25,000 rpm (Beckman L7-65 ultracentrifuge with SW-28 rotor) for 2h at 4°C using a 30% sucrose cushion solution (30% sucrose dissolved in 100 mM NaCl, 10 mM Tris-HCl, 1mM tris-ethylenediaminetetraacetic acid [EDTA] buffer; pH 7.4). The supernatant was then aspirated, and the virus resuspended in 2mL of phosphate buffered saline (PBS, pH7.4). The purified virus preparation was then inactivated using neutral buffered 0.03% formalin for 48h at 4˚C.

### Capture monoclonal antibodies

Murine monoclonal antibodies (mAbs) 1G4 (directed against the H4 head domain), 1H4 (directed against the H5 head domain), 1A7 (directed against the H8 head domain), 2C2 (directed against the H11 head domain), and 1H2 (directed against the H12 head domain) were generated using a classical hybridoma fusion protocol as described in detail in [19]. Briefly, BALB/c mice were immunized intraperitoneally (under ketamine (0.15mg/kg)-xylazine (0.03mg/kg) anesthesia) with 10^5^ PFU of one of the following viruses diluted in 100 μl of PBS: cH5/1_PR8_N1 (H5 head domain from VN04 and stalk domain as well as NA and internal genes from PR8), cH8/1_Cal09_N1 (H8 head domain from mSW02, H1 stalk domain and NA from Cal09 and internal genes from PR8), cH11/1_Cal09_N1 (H11 head domain from sNL99 on top of a Cal09 H1 stalk domain, NA from Cal09, and internal genes from PR8) or cH12/1_Cal09_N1 (H12 head domain from mIA07 on top of an H1 Cal09 stalk domain, NA from Cal09, and internal genes from PR8) [17, 18]. Four weeks later mice were infected intranasally with the same virus dose diluted in 50 μl PBS. Three to six weeks post infection one mouse per virus was injected intraperitoneally with 100 μg of the respective purified virus adjuvanted with 10 μg of polyinosinic:polycytidylic acid (Invivogen). Three days post infection spleens were harvested and the hybridoma fusion was performed according to the published protocol [19]. Hybridoma clones were then screened for reactivity against the respective recombinantly expressed HA proteins [12, 20] via ELISA and Western blot (16). MAbs were further characterized using the HI assay and immunostaining of cells infected with the respective virus. Finally, mAbs 1H4, 1A7, 2C2, and 1H2 were selected due to their reactivity in all assays including activity Western blot under reducing and denaturing conditions which indicates binding to a linear epitope. Generation and properties of mAbs 7B2 (directed against the HA head domain Cal09-like pandemic H1N1 viruses) and PY102 (directed against the HA head domain H1N1 strain PR8) are described elsewhere [14, 21]. Hybridomas were produced and purified via a protein G column using a standard protocol [14]. All animal procedures were prospectively approved and performed in accordance with the Icahn School of Medicine at Mount Sinai Institutional Animal Care and Use Committee (IACUC).

### Biotinylation of detection mAbs

Stalk-reactive human mAb CR9114 was purified from transfected cells, while murine mAbs KB2 and 6F12 were purified from hybridoma supernatants via a protein G column using a standard protocol [14, 22, 23]. Biotinylation of murine mAbs KB2 and 6F12 was performed using the EZ-link NHS-PEG4-Biotin kit (Thermo Fischer Scientific). Briefly, antibodies were biotinylated using a 20-fold molar excess of biotin. The antibody-biotin mixture was incubated at room temperature for 30min, and then filtered through a Zebra Spin desalting column by spinning at 1500rcf for 1 min (Thermo Fisher Scientific).

### Testing KB2 binding to HA treated at low pH with/without reducing agent

Microtiter 96-well plates (Immulon 4HBX; Thermo Fisher Scientific) were coated with 5μg/mL of a purified preparation of cH5/1_Cal09_N1 virus (HA head domain from VN04, HA stalk domain and NA from Cal09, internal genes from PR8—see Table 1). The purified virus was diluted in coating solution (KPL) and the plates were then incubated overnight at 4°C. The next day, plates were washed three times with PBS containing 0.1% Tween 20 (PBS-T). The plates were blocked for 1h at 20°C with blocking solution (3% goat serum (Life Technologies, Inc.) and 0.5% milk powder (Thermo Fisher Scientific). Blocking solution was discarded and plates were incubated with different pH-buffered solutions (pH4.4 or pH7 0.1M citric acid/0.2M Na_2_HPO_4_ buffered solution). Low pH treatment was also combined with 0.2M dithiothreitol (DTT) to reduce disulfide bonds. Plates were incubated with pH buffered solutions for 1h at 20°C. Then, the plates were washed three times with PBS-T and followed by incubation with mAb KB2. The mAb was initially diluted to 30μg/mL and then serially diluted in 1:3 steps in blocking solution. After a 2h incubation at 20°C the plates were washed three times with PBS-T and incubated for 1h at 20°C with anti-mouse IgG specific antibody conjugated to horseradish peroxidase (HRP) (Rockland Immunochemicals Inc.) at a 1:3000 dilution. Following four washes with PBS-T, the plates were developed with SigmaFast o-phenylenediamine dihydrochloride (OPD; Sigma) for 10mins. The development was stopped with 3M hydrochloric acid and plates were immediately read at an optical density (OD) of 490nm using a Synergy H1 hybrid multimode microplate reader (BioTek).

### Protein standards

Recombinant protein standards were produced using the baculovirus expression system in insect cells as described before [12, 20]. H1 HA from Cal09 was used as a reference standard for both Cal09 and DR13 viruses. cH5/1_Cal09ss_, cH8/1_Cal09ss,_ cH11/1_Cal09ss_, cH12/1_Cal09ss_ proteins were used as reference standards for cH5/1N1, cH8/1N1, cH11/1N1, and cH12/1N1 viruses respectively [24, 25]. The E374G stalk stabilizing (ss) mutation was present in the stalk domain of all recombinantly expressed chimeric HAs [26].

### Escape mutant generation

The KB2 escape mutant of NL09 was generated similarly to methods described previously [27, 28]. A monolayer of MDCK cells grown in a sterile 6-well cell culture plate (Sigma), was infected with virus at a multiplicity of infection (MOI) of 0.01 along with 0.25x the 50% inhibitory concentration (IC_50_, the IC_50_ of mAb KB2 is approximately 13 μg/mL) of KB2 in 1X minimal essential medium (MEM; 10% 10X MEM, 1% 200mM L-glutamine, 1.6% sodium bicarbonate stock solution (7.5%), 1% stock solution 4-(2-hydroxyethyl)-1-piperazineethanesulfonic acid [HEPES, 1M], 1% antibiotic mix (100 U/ml penicillin-100 μg/ml streptomycin; Gibco), 0.6% bovine serum albumin (BSA) stock solution (35% w/v)) containing TPCK-trypsin (1 μg/mL) for 48h at 37°C and 5% CO_2_. Additionally, a monolayer was infected with virus and an irrelevant control antibody that targets the Ebola virus glycoprotein (mAb 1C12)[29]. After 48h, cells were checked for signs of cytopathic effects (CPE) from virus replication. If CPE was detected, the supernatant from the cells was collected, spun down for 5 minutes at 13,000rpm (to remove cells) and added to a new monolayer of MDCK cells with 0.5x IC_50_ of KB2 (or 0.0425 mg/mL of 1C12 for the control passage). Passages continued until 8x IC_50_ of KB2 (KB2 final concentration of 104 μg/mL) or 100 μg/mL of 1C12 were reached. Final passages of the viruses were collected and plaque purified to create monoclonal stocks for deep sequencing.

### Immunofluorescence staining

To verify that the observed mutation was responsible for loss of binding by KB2, MDCK cells were plated in a 96-well, sterile, flat bottom tissue culture plate (Sigma) and subsequently infected with wild type NL09 virus, plaque purified NL09 virus which had been passaged in the presence of mAb KB2, or plaque purified NL09 virus passaged with control mAb 1C12 at an estimated MOI of 10. Mock-infected cells were used as an additional control. After incubation for 18h at 37°C in MEM lacking TPCK-trypsin (allowing only single cycle virus replication), medium was removed and the cells were fixed with 3.7% paraformaldehyde (PFA) at room temperature for 1h. The PFA was discarded and replaced with 3% non-fat milk in PBS for 1h. For the primary antibody step, plates were incubated with either KB2 at 30μg/mL, or positive control antibodies (a polyclonal cocktail of mouse sera raised against pandemic H1N1 virus) at a 1:100 dilution in 1% non-fat milk (100μL/well) for 1h at room temperature while slightly shaking. Plates were washed three times with PBS and incubated with Alex Flour 488 conjugated goat anti-mouse secondary antibody in 1% non-fat milk (100μL/well) for 1h at room temperature in the dark, while shaking. Finally, after washing three additional times with PBS, cells were visualized using fluorescent microscopy (EVOS XL cell imaging system, Life technologies, Inc.).

### Three-dimensional mapping of escape mutation

The KB2 escape mutation was represented on a three-dimensional structure of the HA of PR8 (PDB ID: 1RU7 [30]) using PyMOL version 1.8.6.2 (Schrödinger).

### Competition ELISA

Plates (96-well; Immulon 4 HBX, Thermo Fisher Scientific) were coated with 2μg/mL (50μl/well) of recombinant cH5/1_Cal09ss_ protein in coating solution (KPL). Plates were incubated overnight and then blocked with blocking solution (3% goat serum (Life technologies, Inc) and 0.5% milk powder in PBS-T) (220μL/well). The plates were blocked for 1.5h at 20˚C and then washed three times with PBS-T. After washing, competing monoclonal antibodies were added (20μg/mL diluted in blocking solution, 100μL/well) following incubation for 2h at 20˚C. After incubating with competing antibodies, plates were washed again three times with PBS-T and then the target biotinylated antibody (KB2) was added. The biotinylated mAb KB2 was first added at a concentration of 30μg/mL and then diluted in 1:3 steps in blocking solution. The plates were incubated for 2h at 20˚C with KB2. After 2h, the plates were washed 3 times with PBS-T and then incubated with streptavidin conjugated to horseradish peroxidase (1:3000, 50 μl per well, Thermo Fisher Scientific). After a 1h incubation at 20˚C, plates were washed 4 times with PBS-T and then developed with SigmaFast o-phenylenediamine dichloride (OPD, 100 μl per well, Sigma) for 10min. The development was stopped with 3 molar hydrochloric acid (50 μl per well). The plates were then read with a synergy H1 hybrid multimode microplate reader (BioTek) at an optical density of 490nm.

### Stability plan

Various storage and treatment conditions for the virus preparations were tested. Day 0 values were measured right after inactivation. A proportion of the preparation was then subjected to pH4 treatment for 60 minutes and then brought back to neutral pH using PBS before the HA concentration measurement. Another proportion was heated to 100˚C for 10 minutes on a heating block, cooled down to 4˚C and was then subjected to HA concentration measurement. In addition, virus preparations (see Table 1) were either stored at 4˚C and HA concentrations were measured on 0, 12, 30, 90, and 180 days or were stored at 27˚C and HA concentrations measured on 0, 12 and 30 days (2 measurements/time points).

### Capture ELISA set up and calculations

Microtiter 96-well plates (Immulon 4 HBX; Thermo Fisher) were coated with 2μg/mL (100μL/well) of capture monoclonal anti-head antibodies as indicated in Table 2. Capture antibodies were initially diluted in carbonate-bicarbonate coating buffer (0.1M Na_2_CO_3_/NaHCO_3_, pH 9.4) for preliminary experiments. Commercially available coating solution (KPL) was used for later experiments. Plates were then stored at 4°C overnight. The following day, plates were washed three times with PBS-T using an automated plate washer (Molecular Devices, Aquamax 2000), and then blocked with PBS-T containing 3% milk (blocking solution, 225μl/well) for 1h at 20°C. In the meantime, purified viral preparations were diluted 1:10 in PBS-T containing 0.05% zwittergent 3–14 (Sigma Aldrich), and protein standards diluted to 16μg/mL in PBS-T containing 0.05% zwittergent 3–14. The dilutions were incubated at room temperature for 1h. The blocking solution was then discarded and the plates were dabbed on a dry paper towel. One hundred μL of test antigen or reference was added to the first well and then serially diluted in 1:4 steps in blocking solution. The plates were incubated for 2h at 20°C and then washed 3x with PBS-T. One hundred μL of biotinylated KB2 detection antibody, diluted to 5μg/mL was then added. In some of the assays mAbs 6F12 (biotinylated) or CR9114 were used as well. Plates were again incubated for 1h at 20°C. Subsequently, plates were washed 3x with PBS-T and 100μL of 1:3000 diluted streptavidin linked to HRP (used for KB2 and 6F12) (Thermo Fisher Scientific) or anti-human IgG (Fab-specific, used for CR9114) HRP (Sigma A0293) were added. After incubating for 1h at 20°C, the plates were washed 4x with PBS-T. After developing for 10min with SigmaFast OPD, the reaction was stopped by addition of 3M hydrochloric acid and read at an absorbance of 490 nm using a synergy H1 hybrid multimode microplate reader (Biotek). The 50% effective concentration (EC_50_) values were calculated using Graphpad Prism 7 to perform the analysis. HA concentrations of the tested samples were calculated in relation to the standard.

**Table 2:** ELISA reagents used for capture ELISA.

Virus	Control Protein	Capture antibody	Primary (detection) antibody (biotinylated)	Secondary antibody
**cH4/3N2**	cH4/3_HK14_	1G4	CR9114 (non-biotinylated)	anti-human IgG HRP
**cH5/1N1**	cH5/1_Cal09ss_	1H4	CR9114 (non-biotinylated), KB2, 6F12	anti-human IgG HRP Streptavidin HRP
**cH8/1N1**	cH8/1_Cal09ss_	1A7	CR9114 (non-biotinylated), KB2, 6F12	anti-human IgG HRP Streptavidin HRP
**cH11/1N1**	cH11/1_Cal09ss_	2C2	KB2	Streptavidin HRP
**cH12/1N1**	cH12/1_Cal09ss_	1H2	KB2	Streptavidin HRP
**Cal09**	Cal09 HA	7B2	KB2	Streptavidin HRP
**DR13**	Cal09 HA	7B2	CR9114 (non-biotinylated), KB2, 6F12	anti-human IgG HRP Streptavidin HRP
**NL09**	Cal09 HA	7B2	CR9114 (non-biotinylated), KB2, 6F12	anti-human IgG HRP Streptavidin HRP
**PR8**	PR8 HA	PY102	CR9114 (non-biotinylated), KB2, 6F12	anti-human IgG HRP Streptavidin HRP

## Results

### Development of a capture ELISA to quantify HA with properly folded stalk domains

**Fig 1:**
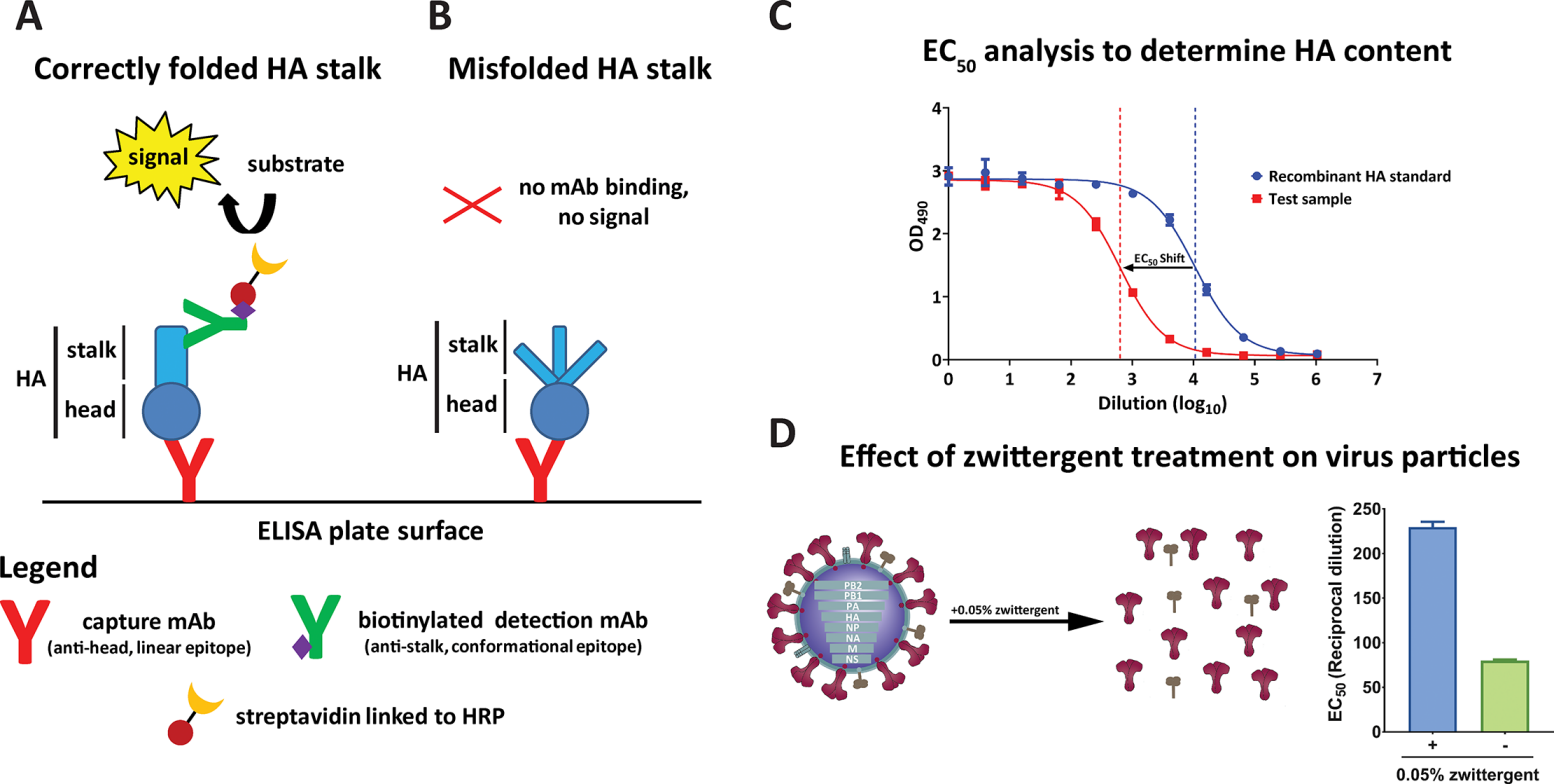
Development of capture ELISA to measure correctly folded HA stalk domain. (**A**) and (**B**) describe the underlying concept of the assay. The assay was primarily developed to detect HA with conformationally intact stalk epitopes. The capture mAb in this assay recognizes the HA head linear epitope. On the other hand, the detection mAb recognizes the conformational epitope in the HA stalk. **(A)** If the stalk of the HA is properly folded, the detection mAb can bind and thus produces a signal. **(B)** If the stalk is misfolded, no signal is observed since the detection mAb cannot recognize the HA stalk. **(C)** HA concentration was calculated using a curve-fit model similar to parallel line analysis in which EC_50_ values are used to determine relative amounts. An EC_50_ analysis is shown using a recombinant protein standard with a known concentration for reference. The shift in EC_50_ between protein standard and test samples was then used to calculate the HA content of test samples. **(D)** The test samples were treated with 0.05% zwittergent for 1h to solubilize the influenza virus membrane and prevent transmembrane-driven rosette formation. EC_50_ values of virus preparations treated with or without zwittergent were calculated. Bars represent mean with error bars representing standard error of the mean.

We initially generated and characterized different murine monoclonal antibodies (mAbs) that bind to epitopes on the globular head domain of different HA subtypes. These mAbs, PY102 (anti-PR8 H1), 7B2 (anti-pandemic H1), 1H4 (anti-H5), 1A7 (anti-H8), 2C2 (anti-H11), and 1H2 (anti-H12) were used for coating ELISA plates to capture HA [14]. Murine mAb KB2 (anti-stalk, binds to H1, H5 and H6), was conjugated to biotin to detect the HA that had been captured. Using KB2, an anti-HA stalk antibody that recognizes a conformational epitope, allowed us to detect HA with a correctly folded stalk domain (Fig 1A). If the stalk is denatured, the detection mAb would not be able to bind, and hence result in a loss of ELISA signal (Fig 1B). A recombinant protein standard with a known concentration was used for reference to allow calculating the HA concentration in the test sample. Briefly, half maximal effective concentrations (EC_50_) for both the recombinant protein standard and test sample were calculated. The shift in EC_50_ values between the protein standard and the test sample was used to quantify the HA content in the test sample (Fig 1C).

In a split vaccine preparation, HA molecules are unlikely to exist as single trimers. The individual molecules usually form rosettes that are associated by hydrophobic interactions of the HA transmembrane domain [31]. The formation of these aggregated rosettes can possibly mask conformational and linear epitopes and thus inhibit mAbs binding. Additionally, the signal may correlate with the number of HA aggregates rather than HA concentration. Therefore, we tested the use of a detergent to dissociate the aggregated HA particle. We used 0.05% zwittergent 3–14, a mild ionic detergent, to permeabilize the membrane and to further disrupt aggregated HA molecules. Addition of zwittergent to the test samples resulted in an increase in the ELISA signal and consequently a higher EC_50_ compared to the non-zwittergent treated test sample (Fig 1D). The use of a detergent also allows for the measurement of HA content in preparations of whole virus or infected cells due to its solubilizing effect on lipid membranes.

### Different anti-stalk detection antibodies produce similar results

**Fig 2:**
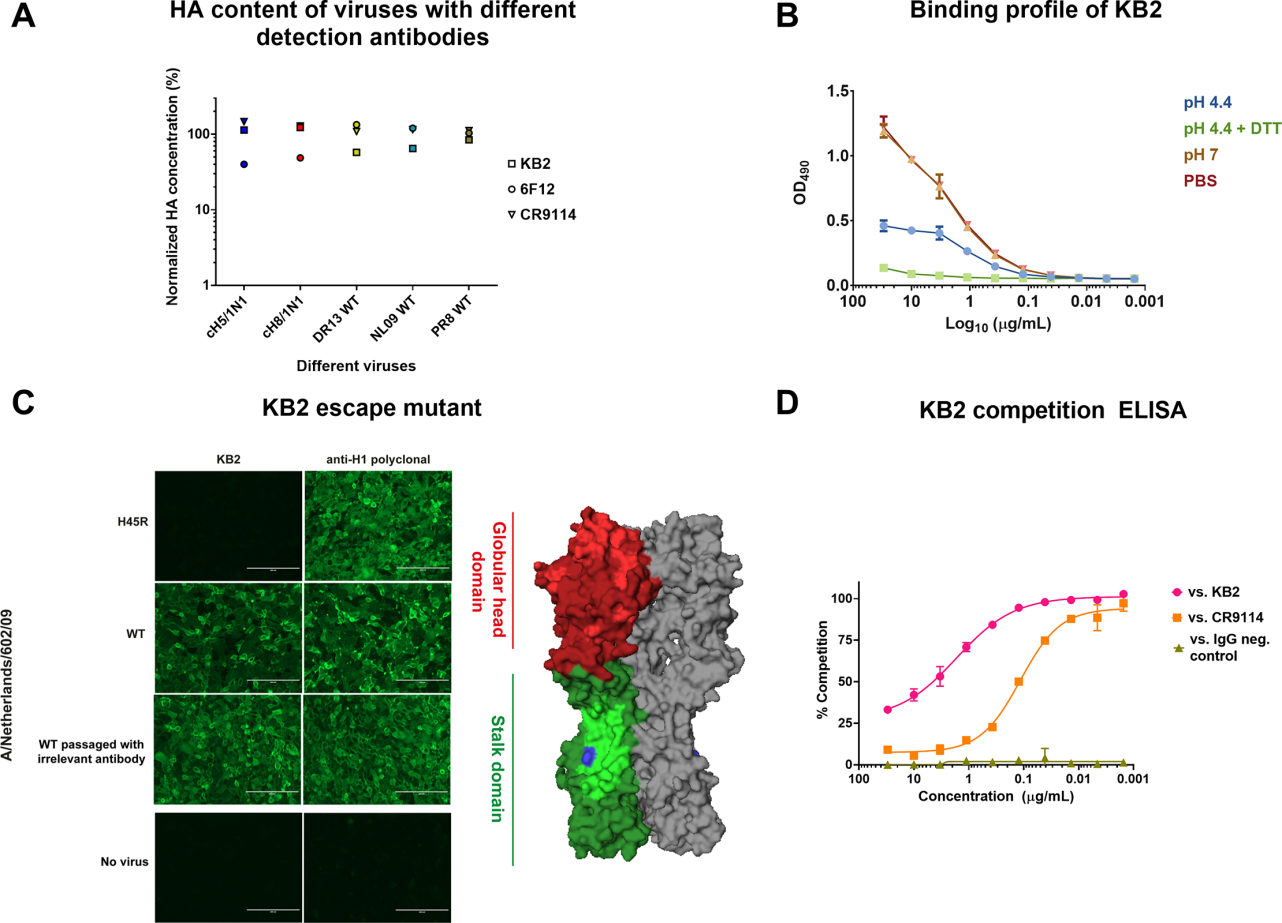
Selection and characterization of the detection mAb. **(A)** Capture ELISAs were performed using three different detection mAbs including murine stalk mAbs KB2 (binds to H1, H5 and H6), 6F12 (pan-H1 binder), and human mAb CR9114 (pan-HA binder). The detection capability of these antibodies was measured using five different purified virus preparations: cH8/1_Cal09_N1, cH5/1 _Cal09_N1, DR13 (pandemic H1N1), NL09 (pandemic H1N1) and PR8 (H1N1). The amount measure with each mAb was normalized to the mean value of all three mAbs and expressed as percent of the mean. KB2 was selected for further assay development and characterized in more detail. (**B**) To understand if KB2 is sensitive to conformational changes of the HA a cH5/1_Cal09ss_N1 purified virus preparation was coated on ELISA plates and exposed to either PBS control, buffered solutions at pH4.4 (with and without reducing agent DTT) or at pH7 for one hour, and finally brought back to neutral pH prior to performing the ELISA. **(C)** Immunofluorescent staining of NL09 wildtype (WT) virus and escape mutant infected MDCK cells was done using KB2 and polyclonal serum against H1. NL09 was also passaged in the presence of irrelevant mAb as control. The right panel shows the structure of HA. Red denotes the globular head domain, while green denotes the stalk domain. Head antigenic sites are denoted in light red, and the highly conserved stalk epitopes are denoted in light green (FI6v3, F10, CR9114, CR6261) [23, 35, 36, 47]. The H45R position in the stalk of the KB2 escape mutant is denoted in blue (PDB 1RU7 [30]). **(D)** Competition ELISA indicates that KB2 and CR9114 compete for the same epitope in the stalk domain of the H1 HA.

The capture ELISA described here requires the use of a detection mAb that binds to the conserved stalk domain of the HA. Several cross-reactive antibodies against the stalk domain have been isolated from mice and humans [15, 32–36]. These antibodies bind broadly and neutralize viruses from many different subtypes. Initially, three different monoclonal antibodies (KB2, 6F12 and CR9114) were used to measure HA content in purified wild type and cHA virus preparations (Fig 2A). KB2, 6F12, and CR9114 showed comparable detection profiles. MAb 6F12 showed lower signal than KB2 and CR9114 when measuring HA from cH5/1_Cal09_N1 and cH8/1_Cal09_N1 viruses and detected wild type HA from PR8 and NL09 H1N1 strains slightly better (Fig 2A). Due to the narrow specificity of mAb 6F12 (pan-H1 but no binding to HAs of other group 1 subtypes) and commercial restrictions regarding the use of mAb CR9114, mAb KB2 was selected for further characterization and assay development [14].

To further examine KB2's sensitivity to conformational changes within the HA, we exposed purified egg grown preparations of cH5/1_Cal09_N1 virus to different pH buffered solutions for one hour prior to performing the capture ELISA. In addition, we used a reducing reagent, DTT, to determine if reducing disulfide bonds in the HA influenced the binding profile. MAb KB2 showed optimal binding at neutral pH but binding was reduced at pH 4.4 (Fig 2B). Reducing disulfide bonds plus low pH abrogated KB2 binding to HA completely.

Finally, to shed more light on the epitope of KB2 we generated escape mutants by passaging a pandemic H1N1 virus isolate in MDCK cells in the presence of increasing amounts of the mAb. The resulting escape mutant had a mutation in the N-terminus of HA1 (H45R), which is located in the stalk domain. This mutation completely abrogated KB2 binding (Fig 2C), confirming that the mAb binds in the stalk domain. In addition, mAb KB2 was competing in a competition ELISA with mAb CR9114, suggesting that the two epitopes might overlap (Fig 2D). Interestingly, we found that mAb CR9114 still binds to the KB2 escape mutant virus (data not shown), suggesting that the epitope overlap is not complete. Of note, anti-stalk mAb 6F12 also lost binding to the KB2 escape mutant.

### Effect of different treatments on assay performance and ELISA potency and evaluating the assay against a group 2 HA

**Fig 3:**
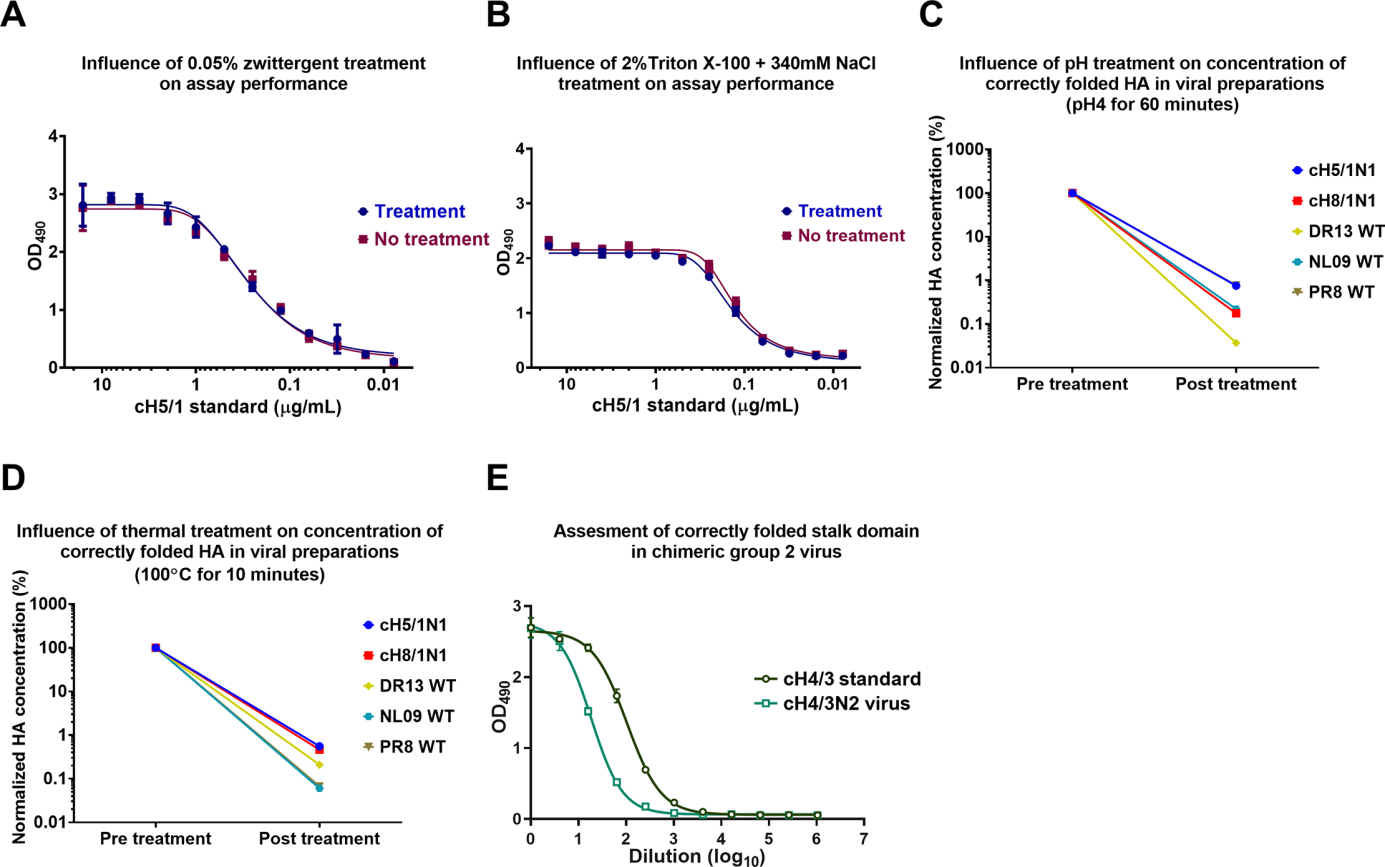
Effect of different stress treatments and group 2 stalk on assay performance. Detergent treatment is necessary for the assay for optimal access antigenic sites on the HA trimers. In addition, conditions during the vaccine production process also require detergent and high salt concentrations. To test if these conditions influence the stalk conformation we treated recombinantly expressed HA protein (which is used as standard in the assay) with **(A)** 0.05% zwittergent and **(B)** 2% Triton X-100+340mM NaCl and compared the readout to the signal obtained with untreated HA. In addition, we tested the influence of different treatments on the concentrations of properly folded HA in purified viral preparations. We exposed the viral preparations to **(C)** low pH (pH 4 for 60 minutes) or **(D)** heat (100°C for 10 minutes) treatment. Post treatment HA concentrations were normalized to pre-treatment concentrations. **(E) A** capture ELISA was performed to measure the HA content of a group 2 cHA expressing virus, cH4/3N2, with a recombinant cH4/3 protein as standard.

The capture ELISA we have developed requires the use of detergents to solubilize HA trimers and prevent HA aggregation or rosette formation. However, it was unclear whether detergent treatment would impact on conformational stalk epitopes. To test this, we measured the signal against the reference antigen (recombinant cH5/1 HA) in its untreated form and when treated with zwittergent. The use of 0.05% zwittergent had no effect on the detection of rHA (Fig 3A). Likewise, the use of a high salt concentration combined with a high concentration of Triton X-100 as used during the influenza virus vaccine production process for splitting virus (2% Triton X-100 + 340mM NaCl) had no effect on the rHA detection and conformation (Fig 3B) [37].

High temperature and low pH conditions are known to affect influenza virus glycoprotein conformation [38, 39]. To investigate if these conformational changes could be detected with our assay, viruses (cH5/1_Cal09_N1, cH8/1_Cal09_N1, DR13, NL09 and PR8) were treated with a pH4 solution for 60 minutes or were heated to 100°C for 10 minutes. After treatment with low pH, most of the signal in the capture ELISA was lost in the case of all five test viruses (Fig 3C). Similarly, heat treatment at 100°C also seemed to destroy most of the HA and thus resulted in a negligible HA detection (Fig 3D). Additionally, we also adapted our capture ELISA method to measure the HA content of a group 2 cH4/3N2 virus preparations (Fig 3E). These data show that the capture ELISA method can discriminate between native and denatured HAs, and can also be used to measure HA content of viruses other than the ones expressing group 1 HA.

### Capture ELISA to measure HA stalk stability in virus preparations over time

We designed a stability study that included several virus preparations that were stored at 4°C and 27°C and sampled over a six-month time period. For this purpose, we used different wild type and chimeric HA expressing viruses (Table 1). H1N1 viruses circulating early in the 2009 pandemic expressed an HA that had an unstable stalk domain [25, 40, 41]. A mutation in position 374 that changed a glutamic acid into a lysine appeared quickly in nature and stabilized the HA [42]. In addition, mutating glutamic acid in position 374 to glycine has been found to be stabilize the HA trimer [26]. The instability of the HA trimer has been implicated as one factor in the recent failure of the H1N1 component of live-attenuated vaccines to induce a protective immune response [24]. Since this mutation could potentially also impact on the stability of inactivated virus vaccines, we constructed viruses expressing chimeric HAs with the head domains of either H5, H8, H11 or H12 and the stalk domains of either Cal09 ('unstable' in the wild type HA), a wild type isolate that possesses the stabilizing mutation (DR13) or Cal09 with a stabilizing E374K mutation (Cal09ss). Wild type viruses tested included pandemic H1N1 strains Cal09 and DR13 (Table 1). The viruses were propagated in embryonated eggs, concentrated using ultracentrifugation, and inactivated using formalin. While these are very crude preparations that do not resemble split virus products, we wanted to assess if trends in stability over time would emerge from different head and stalk combinations. The HA content of virus preparations stored at 4°C was assessed at days 0, 12, 30, 60, 90, and 180 while preparations stored at 27°C were sampled on days 0, 12, and 30.

**Fig 4:**
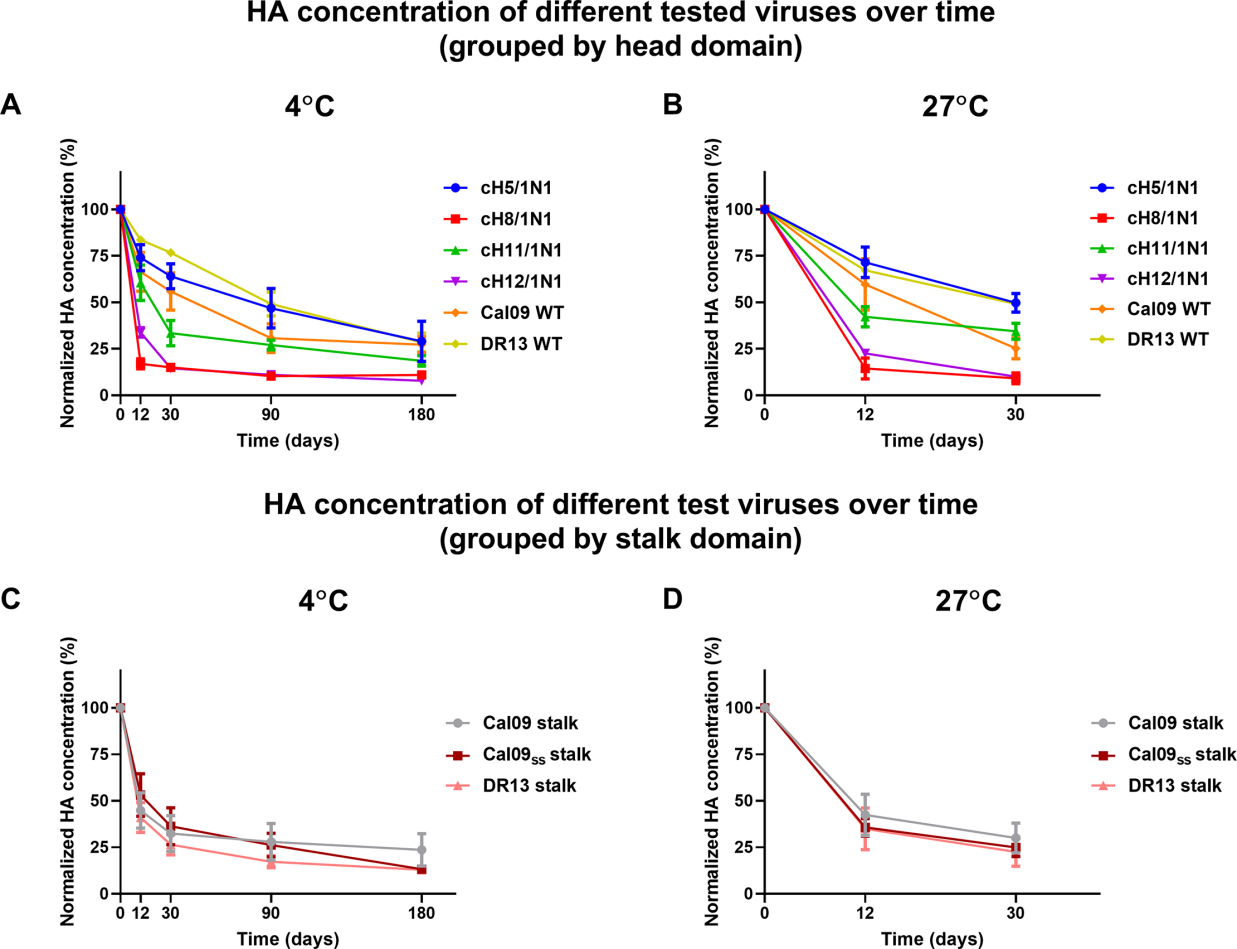
Stalk stability under different storage conditions. HA content of crude cH5/1N1, cH8/1N1, cH11/1N1, cH12/1N1, wild type Cal09 and wild type DR13 preparations at 4°C and 27°C. The HA content of samples stored at 4°C was measured on days 0, 12, 30, 90 and 180; and HA content of samples stored at 27°C was measured on days 0, 12 and 30. In order to better understand the effects of the storage conditions on the stability of the two main HA domains (head and stalk), viruses were grouped based on either **(A, B)** head domain or **(C, D)** stalk domains. Data points in A and B represent an average of two replicates for the wild type strains and an average of three viruses (Cal09, Cal09ss, DR13) for cHA strains, while the data points in C and D represent an average of five viruses (cH5/1N1, cH8/1N1, cH11/1N1, cH12/1N1 and wild type) for Cal09 and DR13 stalk viruses and four viruses (cH5/1N1, cH8/1N1, cH11/1N1, cH12/1N1) for the Cal09ss stalk. The error bars represent the standard error of mean. All HA concentrations were normalized to concentrations measured on day 0.

As expected, all virus preparations lost HA content over time at both temperatures. To make thee analysis easier we grouped viruses first by their head domains and then by their stalk domains. When grouped by the different head domains, we found that constructs that expressed the H5 head domain were most stable and were comparable to the DR13 wild type virus at both temperatures (Fig 4A and 4B). Cal09 wild type virus was less stable than DR13, confirming earlier findings. The H11 head domain also conferred stability while virus preparations with both the H8 and H12 head domains lost HA content relatively quickly. These findings held true at both temperatures (Fig 4A and 4B). No trends were seen when the chimeric HA expressing viruses were grouped by stalk domain (Cal09, Cal09ss and DR13) (Fig 4C and 4D). Importantly, while trends for each construct in split vaccine preparations might be similar, the rapid loss of HA content seen in these experiments might be the result of the crude nature of the antigen preparation.

## Discussion

The single radial immunodiffusion (SRID) assay, which was developed in the early 1970’s, has been considered the gold standard to measure the HA concentration in vaccine preparations [1, 2]. We have developed a capture ELISA that can also measure the HA content of antigen/vaccine preparations. However, unlike SRID assays, the capture ELISA method is able to discriminate between HAs with native and denatured HA stalk domains. The methodology of this ELISA is based on binding the HA to a plate using capture antibodies that are directed towards the head domain. An antibody against the stalk domain is then used to detect the HA. We tested several anti-stalk antibodies in the assay and chose mAb KB2 for further development. KB2 is a neutralizing mAb that is protective *in vivo* and binds to group 1 stalk domains including H1, H5, and H6. Its epitope is sensitive to conformational changes including those induced by low pH treatment or reducing conditions [17, 22, 43]. Therefore, only correctly folded HA will be detected in the assay. Importantly, detergent treatment with Triton X-100 at high salt concentrations–a condition used during the virus splitting process in vaccine manufacturing—did not negatively impact the performance of the assay. Importantly, while our effort focused on mAb KB2, there might be value in developing a panel of mAbs to be used in this assay set-up. Different anti-stalk mAbs have different binding footprints and angles of approaches and might therefore have different sensitivities as shown above with 6F12 in comparison to KB2 and CR9114.

We have also implemented our capture ELISA methodology to measure HA stalk conformation of different chimeric HA-expressing viruses. Chimeric HA-based vaccine strategies have been shown to induce broadly protective stalk specific antibodies in different animal models and have entered clinical development [8, 18, 44–46]. It is therefore important to develop a method that is suitable to measure the concentration of correctly folded HA stalks in vaccine preparations that are intended to induce broadly protective, conformation dependent anti-stalk antibodies. This also applies to other vaccine approaches that are based on raising immunity towards the conserved stalk domain such as the use of headless HA constructs [10, 11].

The instability of the stalk of the 2009 pandemic H1N1 HA has been speculated to be the cause of low vaccine efficacy of post-pandemic LAIV preparations [24]. The HA can be stabilized by replacing the glutamic acid in position 374 with a lysine or glycine [24, 26, 42]. A stabilizing E374K mutation has also been detected in circulating pandemic H1N1 strains as early as 2009 and this mutation is now found in the majority of isolated strains [42]. Furthermore, it has been determined that, even though there are other amino acid changes between pre and post pandemic strains, the E374K mutation alone is responsible for the enhanced stability of the HA [42]. We tested if this mutation could also have a positive impact on stability of chimeric HA expressing virus preparations. While this mutation ultimately had no impact on the stability, our results indeed indicate that the choice of the head domain influences stability greatly. Constructs containing H5 and H11 head domains displayed greater stability than constructs containing H8 and H12 head domains. While it is important to stress that these results are based on crude, research grade virus preparations that do not support virus stability very well over time, further studies may confirm that the choice of head/stalk combinations are of importance for stability of cHAs. This would not be surprising since the inter-subunit interactions between the head domains of certain influenza subtypes/strains (e.g. pre-pandemic human H1N1) have been shown to be important for trimer stability [42]. We also found that a later pandemic H1N1 isolate, DR13 (which features K374) showed higher stability than the earlier Cal09 isolate (E374) confirming the importance of the amino acid in position 374 in determining the HA stability. Here, we were primarily interested in measuring the concentration of correctly folded stalk domain of cHAs. However, the assay might be further developed in the future to measure the concentration of HA with correctly folded stalk domains in currently available seasonal influenza virus vaccines as well.

In summary, we have developed an ELISA to quantitatively measure the concentration of HA with correctly folded stalks in vaccine preparations. While we mostly focused on group 1 stalk domains, we show that a similar assay can be used for group 2 HAs as well. In addition, this methodology might be further expanded to influenza B HAs. It is conceivable that further optimization of the assay setup includes multiple stalk-reactive detection antibodies which could make the assay more robust. This ELISA assay should support the development of new types of broadly protective/universal influenza virus vaccines and could be established as potency assays for these stalk-based vaccine constructs. However, the establishment of a true potency assay will require expanded studies establishing a correlation between concentrations measured in the assay and immunogenicity in animal models.
